# Crohn's disease and ulcerative colitis in β-thalassemia major: A report of two cases and a brief review of the literature 

**DOI:** 10.3892/mi.2026.331

**Published:** 2026-07-22

**Authors:** Trwska A. Amin, Luqman K.H. Rasool, Choman H. Abdulqadir, Shaho F. Ahmed, Rawa M. Ali, Karokh H. Salih, Dana T. Gharib, Hoshmand R. Asaad, Hiwa O. Abdullah, Fahmi H. Kakamad, Zana B. Najmadden

**Affiliations:** 1 Department of Hematology, Thalassaemia and Congenital Blood Diseases Center, Sulaymaniyah 46001, Iraq; 2 Department of Endocrinology, Smart Health Tower, Sulaymaniyah 46001, Iraq; 3 Department of Pathology, Smart Health Tower, Sulaymaniyah 46001, Iraq; 4 Department of Pathology, Hospital for The Treatment of Chemical Weapons Victims, Halabja 46018, Iraq; 5 Department of Basic Medical Sciences, College of Medicine, University of Sulaimani, Sulaymaniyah 46001, Iraq; 6 Department of Gastroenterology and Hepatology, Smart Health Tower, Sulaymaniyah 46001, Iraq; 7 Department of Gastroenterology, Gastroenterology and Hepatology Teaching Hospital, Sulaymaniyah 46001, Iraq; 8 Department of Scientific Affairs, Smart Health Tower, Sulaymaniyah 46001, Iraq; 9 Kscien Organization for Scientific Research, Sulaymaniyah 46001, Iraq; 10 Department of Clinical Sciences, College of Medicine, University of Sulaimani, Sulaymaniyah 46001, Iraq; 11 Research Center, University of Halabja, Halabja 46018, Iraq

**Keywords:** β-thalassemia major, inflammatory bowel disease, Crohn's disease, ulcerative colitis, adolescent

## Abstract

The co-occurrence of β-thalassemia major and inflammatory bowel disease is exceedingly rare, yet clinically important, as overlapping manifestations such as severe anemia, fatigue, abdominal pain and dysentery may mask the underlying inflammatory bowel disease and delay diagnosis. The patients described in the present case report exhibited markedly elevated levels of inflammatory markers and fecal calprotectin levels (>1,000 µg/g); these findings, together with colonoscopic and histopathological findings, enabled the diagnosis of both major inflammatory bowel disease subtypes, namely Crohn’s disease and ulcerative colitis. These cases add to the limited literature available by demonstrating that inflammatory bowel disease should be considered in patients with β-thalassemia major presenting with persistent gastrointestinal symptoms, particularly when symptoms are disproportionate to baseline disease manifestations. Early clinical suspicion, prompt diagnostic evaluation, and multidisciplinary management are crucial for preventing further clinical deterioration, minimizing complications and improving long-term outcomes. However, further studies are warranted to clarify the potential pathophysiological association and establish optimal management strategies for this rare coexistence.

## Introduction

Inflammatory bowel disease (IBD) is a chronic inflammatory disorder of the gastrointestinal tract that involves intricate interactions among environmental, immunological, microbiological and genetic factors (1). The disease is classified into Crohn's disease (CD) and ulcerative colitis (UC) (1). As the disease progresses, patients may exhibit several systemic manifestations secondary to an inflammatory process. Anemia, particularly in patients with CD, is a common extraintestinal complication of IBD (1). Anemia in teenagers aged between 12 and 15 years is characterized by a hemoglobin concentration <120 g/l (2). This condition can arise from various causes, including chronic illnesses, deficiencies in iron, vitamin B12, or folate and genetic factors (1,3). The common causes of anemia in IBD can include malnutrition, malabsorption and micronutrient deficiency. However, gastrointestinal bleeding can trigger or aggravate the condition (1). β-thalassemia refers to a group of inherited blood disorders characterized by abnormalities in the production of β chains of hemoglobin. These anomalies result in a wide range of phenotypes, ranging from asymptomatic or mild anemia to severe anemia (3). There are three primary forms of β-thalassemia: Thalassemia major, thalassemia intermedia and thalassemia minor. Thalassemia major is typically diagnosed within the first 2 years of life and is characterized by severe anemia, necessitating regular blood transfusions (3). Due to the rarity of this coexisting condition (IBD and β-thalassemia), the exacerbation of β-thalassemia due to IBD remains poorly understood, as only a few cases have been reported in the literature to date (4-6). The present study reports 2 cases of CD and UC in 2 adolescent patients with β-thalassemia.

## Case report

### Case 1. Patient information

A 15-year-old male presented to the Internal Medicine Clinic of the Thalassaemia and Congenital Blood Diseases Center (Sulaymaniyah, Kurdistan Region, Iraq) on September, 2021 with complaints of colicky abdominal pain, episodic severe dysentery (>10 times a day) for a period of 6 years, and fatigue for 2 months. He had been receiving regular blood transfusions since he was diagnosed with thalassemia major at 1 year of age. Chelation therapy had been required since the age of 5 years. When the patient was 8 years of age, he experienced intermittent abdominal pain, dysentery and weight loss. A general stool examination (GSE) was performed at that time and revealed *Entamoeba histolytica* (*E. histolytica*) infection. The patient received metronidazole. However, 4 years thereafter, the patient was diagnosed with growth failure and treated with growth hormone (GH) replacement therapy for 1 year, although there was no response to the treatment.

*Clinical findings*. Upon a physical examination, he had a typical thalassemic face (frontal bossing and flattened nasal bridge), jaundice, tachycardia (110 bpm) and a slightly distended abdomen.

*Diagnostic assessment*. Blood analyses revealed severe anemia (hemoglobin level, 50 g/l), an elevated C-reactive protein (CRP) level (265 mg/l), a high erythrocyte sedimentation rate (120 mm/h) and a negative Coombs test. He had an infection with *E. histolytica* according to the GSE, and the fecal calprotectin level was elevated (>1,000 µg/g). The patient was referred to a pediatric gastroenterologist, who performed a colonoscopy and obtained biopsies. The histopathological analysis of the specimens confirmed the diagnosis of CD (data not shown as images could not be retrieved).

*Therapeutic intervention*. The patient was treated with mercaptopurine (25 mg/day), mesalazine (2 g/day, suppository) and prednisolone (20 mg/day). After 1 month, the patient developed cholestatic jaundice, and the use of mercaptopurine was terminated; however, the patient's condition worsened. A magnetic resonance cholangiopancreatography scan revealed sclerosing cholangitis (Fig. 1), and he was treated with ursodeoxycholic acid (13-15 mg/kg/day, divided into 2-3 doses for a lifetime duration).

*Follow-up*. The patient remained under follow-up for the monitoring of cholangitis. The frequency of diarrhea decreased to three to five episodes per day, and the hemoglobin level increased to 80 g/l. Unfortunately, the patient's cholangitis progressively worsened, leading to jaundice and sepsis. Despite treatment, the patient's condition continued to deteriorate, and the patient succumbed ~2 years after the initial presentation.

### Case 2. Patient information

A 13-year-old female patient presented with thalassemia major to the Thalassaemia and Congenital Blood Diseases Center (Sulaymaniyah, Iraq) on September, 2021 with severe dysentery (more than 8 times/day), colicky abdominal pain, fatigue and increasing pallor. The patient had been on regular transfusions since 3 months of age and commenced chelation at the age of 3 years. At the age of 11 years, she was diagnosed with growth failure and underwent 1 year of GH replacement therapy without significant improvement. She reported a history of intermittent abdominal pain, weight loss and bloody diarrhea for 3 years. On each occasion, GSE tested positive for *E. histolytica*, for which she had been prescribed antibiotics by a gastroenterologist. Recently, the patient's condition had begun to deteriorate further.

Clinical findings. A physical examination revealed that she had a typical thalassemic facial appearance characterized by frontal bossing, a flattened nasal bridge and jaundice.

*Diagnostic assessment*. Blood analyses revealed severe anemia (hemoglobin level, 55 g/l), elevated CRP (360 mg/l) and elevated serum ferritin (4,800 µg/l) levels, with a negative Coombs test. A GSE confirmed an infection with *E. histolytica*. Furthermore, the fecal calprotectin level was high (>1,000 µg/g). A colonoscopy was performed, biopsy samples were obtained, and histopathological analysis was performed on 5-µm-thick paraffin-embedded sections. The sections were fixed in 10% neutral buffered formalin at room temperature for 24 h and subsequently stained with hematoxylin and eosin (Bio Optica Co.) for 1-2 min at room temperature. The sections were then examined under a light microscope (Leica Microsystems GmbH). The histopathological analysis confirmed the diagnosis of UC (Fig. 2).

*Therapeutic intervention*. The case was managed with prednisolone (20 mg/day), mesalazine (500 mg tablet, 1x3) and mercaptopurine (50 mg/day). The chelation therapy was switched to Desferal (deferoxamine) due to the exacerbated diarrhea of the patient caused by Exjade.

*Follow-up*. The patient responded well to the treatment course. Her diarrhea decreased to one to two times/day without containing blood, resulting in an increase in her hemoglobin level to 88 g/l. Stool calprotectin and serum ferritin levels decreased to 75 µg/g and 3,083 µg/l, respectively.

## Discussion

CD is a chronic inflammatory disorder characterized by transmural inflammation in the gastrointestinal tract, resulting in relapsing and remitting symptoms. Approximately half of patients with CD may experience complications over time due to inflammation, including strictures, fistulas, abscesses, or lower gastrointestinal bleeding (7). The disease is associated with various symptoms, such as abdominal pain, diarrhea, weight loss, fever, fatigue, uveitis, arthritis, pyoderma gangrenosum and aphthous stomatitis. However, anemia is the most prevalent systemic complication (6,8). CD can affect both males and females equally at any age, although it is most commonly diagnosed between the ages of 15 and 35 years, and between 55 and 65 years of age (6).

UC is another chronic inflammatory condition primarily affecting the colon, often spreading continuously to involve the entire large intestine. This disorder is characterized by episodes of exacerbation and periods of remission. Both CD and UC are collectively referred to as IBD (1). They may be misdiagnosed with each other due to sharing similar symptoms (9). The sex predilection and age of onset in UC are similar to those in CD. Of note, ~20% of patients with UC have a family member or close relative with UC or CD (9). It has been revealed that ~6% of children are susceptible to the onset of a chronic disease or malignancy during their adolescent years. However, anticipating the likelihood that children will develop a confluence of chronic diseases during adolescence poses a considerable challenge (5). The coexistence of β-thalassemia and IBD is an exceedingly rare occasion, and the understanding of the outcomes of patients affected by both conditions remains limited (4).

According to the available literature, only a small number of case reports to date have highlighted outcomes in cases where IBD coexists with β-thalassemia (5,6,10). The present study reports two rare cases of CD and UC in two adolescent patients with β-thalassemia major. The first case was that of a 15-year-old male who presented with colicky abdominal pain, severe dysentery and fatigue. The second case was that of a 13-year-old female. Her complaints were severe dysentery, colicky abdominal pain and fatigue.

Similar to the present study, Bank and Busari (5) reported the coexistence of CD, autoimmune thyroiditis and β-thalassemia trait in a 15-year-old girl. The patient in their study suffered from abdominal discomfort, fatigue, diarrhea and perirectal bleeding. They reported that the iron-deficiency anemia in their patient may be attributed to the combined effects of chronic perirectal bleeding and β-thalassemia. These factors likely accounted for the pallor and fatigue of the patient. The abdominal pain and perirectal bleeding, on the other hand, were a direct consequence of the pancolitis (5). In the cases described herein, severe anemia may result from β-thalassemia, amebiasis or severe dysentery. As in the previous study (5), fatigue in the cases described herein may be due to anemia. Baş and Özlü (10) also reported sickle cell anemia, β-thalassemia and CD in a 37-year-old female patient. However, they mainly focused on using epidural anesthesia for a laparoscopic cholecystectomy rather than β-thalassemia and CD. The prevalence of anemia is approximately between 6 and 74% in patients diagnosed with IBD (11,12). The two most common subtypes of anemia in IBD are anemia of chronic disease and iron deficiency anemia. According to a previous cross-sectional study, the β-thalassemia trait was found in 6.7% of patients with IBD, with a higher prevalence observed among patients with CD compared to patients with UC (13). The presence of both β-thalassemia trait and IBD can potentially aggravate anemia, requiring careful evaluation, particularly in cases where conventional iron replacement therapy does not yield satisfactory results (4). There are limited clinical reports available addressing the outcomes of patients with IBD with β-thalassemia; however, some of these reports suggest that the coexistence of these conditions may exacerbate disease activity or increase the risk of colon cancer (4-6,14).

Borody *et al* (15), by reporting two cases of CD due to amebiasis, indicated that pathogens can cause the endoscopic and clinical features of the disease. They further reinforced their claim by successfully managing the cases with antibiotics (15). The presenting symptoms in their cases were abdominal pain, fatigue, bloody diarrhea and weight loss. It has been revealed that amebiasis is more common in patients with IBD, particularly in patients with UC, than in the normal population (9). Both cases described herein had a positive history of amebiasis more than once, and their symptoms were the same as those described in the study by Borody *et al* (15). The infection with *E. histolytica*, in addition to chronic diseases, could further aggravate the condition in the cases described herein.

The early diagnosis of IBD is crucial to avoid complications and surgical intervention, and improve the quality of life. While colonoscopy is the most effective diagnostic test for evaluating colitis in asymptomatic individuals, high costs and invasiveness are its major drawbacks. Fecal calprotectin has proven to be a valuable surrogate marker for predicting the clinical recurrence of IBD. However, its potential as a screening tool for the early detection of IBD remains to be determined. Furthermore, anemia may be a critical marker for the early diagnosis of IBD; however, it remains unknown whether it plays the same role in asymptomatic cases (1). In patients with coexisting β-thalassemia trait and IBD, iron supplementation may be necessary due to significant blood loss and severe iron depletion (5). Typical treatment approaches for patients with CD include sulfasalazine derivatives, such as 5-aminosalicylic acid (5-ASA), immunosuppressants, oral steroids, anti-tumor necrosis factor-α therapy and surgery. Conservative treatment is usually applied in an order beginning from agents that are deemed safer, such as 5-ASA, to those with a better response, but with increased side-effects, such as steroids and immunosuppressive drugs (6). In the present case report, the first case was treated with mercaptopurine, mesalazine and prednisolone. The patient developed sclerosing cholangitis, and he was prescribed ursodeoxycholic acid. Following the treatment interval, the patient's condition improved, with a decrease in the frequency of diarrhea and an increase in his hemoglobin level. Despite that, the patient succumbed almost 2 years after presentation due to the aggravation of sclerosing cholangitis. The second case received prednisolone, mercaptopurine and mesalazine, and she responded well to the treatment. The absence of growth data at the time of presentation and the lack of imaging analyses to assess the gastrointestinal tract may represent limitations of the present case report. In addition, the findings may have limited generalizability due to the sample size and study design. Furthermore, the underlying pathophysiology of the coexistence remains unclear and warrants further rigorous investigation.

In conclusion, IBD should be considered in patients with β-thalassemia major presenting with persistent gastrointestinal symptoms, particularly when symptoms are disproportionate to baseline disease manifestations. Early clinical suspicion, prompt diagnostic evaluation and multidisciplinary management are crucial for preventing further clinical deterioration, minimizing complications and improving long-term outcomes. Further studies are warranted to clarify the potential pathophysiological association and establish optimal management strategies for this rare coexistence.

## Figures and Tables

**Figure 1:**
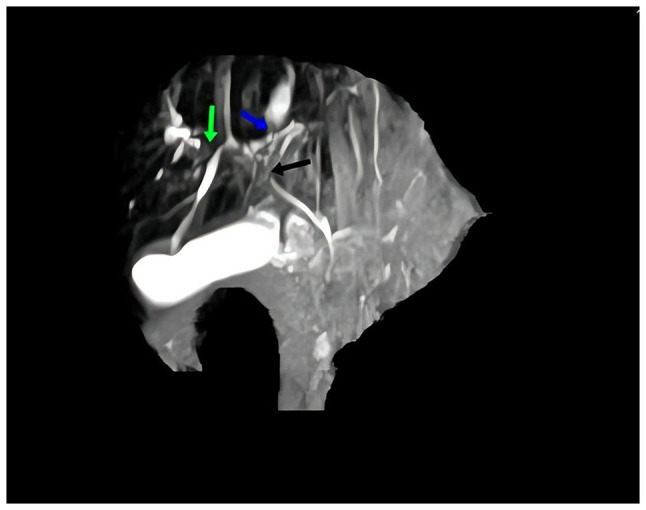
Coronal maximum intensity projection image of magnetic resonance cholangiopancreatography illustrating biliary strictures in the right posterior sectoral duct (green arrow), strictures in the left sectoral duct (blue arrow), and in the upper part of the common bile duct (black arrow).

**Figure 2:**
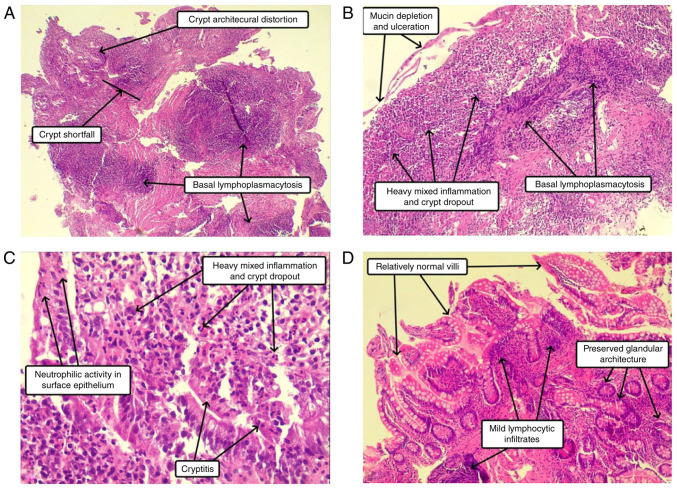
Results of histopathological analysis (hematoxylin and eosin staining). (A) Colonic mucosa with a heavy lymphoplasmacytic infiltrate that extends into the muscularis mucosa, resulting in crypt shortening. The glands vary in size, shape, spacing and orientation. (B) Image illustrates prominent mucin depletion of the surface epithelium with ulceration. There is a heavy mixed inflammatory infiltrate in the lamina propria that extends deeply, with no identifiable viable crypts. (C) Image illustrates neutrophilic activity in the surface epithelium, lamina propria and crypts, associated with a significant reduction in crypt count. (D) Image illustrates the terminal ileum with relatively preserved villous and glandular architecture and patchy lymphocytic infiltrates. The magnification of the images is as follows: (A) x40, (B and D) x100, and (C) x400.

## Data Availability

The data generated in the present study may be requested from the corresponding author.
